# Expression of Hepatocyte Growth Factor-Like Protein in Human Wound Tissue and Its Biological Functionality in Human Keratinocytes

**DOI:** 10.3390/biomedicines3010110

**Published:** 2015-02-04

**Authors:** James C. Glasbey, Andrew J. Sanders, David C. Bosanquet, Fiona Ruge, Keith G. Harding, Wen G. Jiang

**Affiliations:** 1 Cardiff China Medical Research Collaborative (CCMRC), Cardiff University–Peking University Cancer Institute, Cardiff University–Capital Medical University Joint Centre Biomedical Research, Cardiff University School of Medicine, Henry Wellcome Building, Heath Park, Cardiff CF14 4XN, UK; E-Mails: jamesglasbey@gmail.com (J.C.G.); sandersaj1@cf.ac.uk (A.J.S.); davebosanquet@hotmail.com (D.C.B.); ruge@cf.ac.uk (F.R.); 2 Department of Wound Healing and Welsh Wound Innovation Centre, Cardiff University School of Medicine, Heath Park, Cardiff CF14 4XN, UK; E-Mail: hardingkg@cf.ac.uk

**Keywords:** hepatocyte growth factor-like (HGFl), macrophage stimulating protein (MSP), wound healing, human keratinocyte, HaCaT

## Abstract

Hepatocyte growth factor-like protein (HGFl) and its receptor, Recepteur d'Origine Nantais (RON), have been implicated in the development of wound chronicity. HGFl and RON expression was detected in acute wound tissue, chronic wound tissue and in normal skin using quantitative polymerase chain reaction (Q-PCR). HGFl and RON expression was also assessed in chronic healing and chronic non-healing wound tissues using Q-PCR and immunohistochemical staining. Expression was similarly detected in the HaCaT immortalized human keratinocyte cell line using reverse transcription polymerase chain reaction (RT-PCR). rhHGFl was used to assess the impact of this molecule on HaCaT cell functionality using *in vitro* growth assays and electric cell-substrate impendence sensing (ECIS) migration assays. HGFl and RON transcript expression were significantly increased in acute wound tissue compared to chronic wound tissue and were also elevated, though non-significantly, in comparison to normal skin. Minimal expression was seen in both healing and non-healing chronic wounds. Treatment of HaCaT cells with rhHGFl had no effect on growth rates but did enhance cell migration. This effect was abolished by the addition of a phospholipase C gamma (PLCγ) small molecule inhibitor. The increased expression of HGFl and RON in acute, healing wounds and the pro-migratory effect of HGFl in an *in vitro* human keratinocyte model, may indicate a role for HGFl in active wound healing.

## 1. Introduction

Cutaneous wound healing is a complex and multifactorial process. Numerous cellular and molecular players contribute to the ordered process of wound healing [1]. A number of local and systemic aberrations of these healing pathways have been implicated in wound chronicity [2]. Chronic non-healing wounds pose a significant problem to European healthcare providers, with an estimated 3.55–3.70 patients per 1000 population currently undergoing wound treatment [3]. Poor wound healing is a source of significant morbidity, with a myriad of psychosocial implications. Research undertaken in our laboratory has focused on wound profiling and therapeutics [2], describing the action of several molecules on the activity of human epidermal cell lines at the wound edge [4,5,6].

Hepatocyte growth factor-like protein (HGFl), also known as macrophage stimulating protein (MSP) [7], was first characterized by its direct and indirect activation of the resident peritoneal macrophage [8]. The Recepteur d’Origine Nantais (*ron*) gene codes the receptor for this molecule (STK) and is activated when HGFl binds to its β-chain [9]. It has since been identified in numerous other physiological processes, including innate immunity [10], hepatocellular regeneration [11], neuroendocrine cell survival [12] and *in utero* development [8,9]. It has also been implicated in malignant transformation and metastatic potential of a number of tissue types—including liver [13], breast [14], colon [15], lung [16] and pancreas [17] and has been mapped in an at-risk subpopulation of inflammatory bowel disease sufferers [18]. Nanney *et al.* were the first to localize HGFl and its precursors to the human wound site [19]. Subsequently, the influence of HGFl on keratinocyte activity has been explored in multiple animal models. An Australasian group mapped the upregulation and co-localization of HGFl and the structurally related protein hepatocyte growth factor (HGF) in a rat model of excisional wound repair, differentiating a sub-role for HGFl in mature macrophage activation [20]. Whilst a 1998 paper by Bezerra *et al.* [21] denounced the role of HGFl in murine wound healing as non-essential in a knockout model, Wang *et al.* reported a definite concentration-dependent increase in primary murine keratinocyte proliferation with the addition of HGFl [22]. The same Chinese group reported the mandatory presence of phosphatidylinositol-3-kinase in HGFl-dependent human epithelial cell migration [23]. Santoro *et al.* postulated that the pro-migratory activity of HGFl in human epidermal wound healing was achieved by regulation of α6β4 integrin and α3β1 integrin in a critical signaling step [24].

HGFl belongs to the kringle protein family [8], which contains the only other recognized scatter factor HGF [25,26]. HGF is a pleiotropic factor effective in tissue homeostasis and response to insult, including tissue repair and epidermal wound healing. HGF and its isoforms act via the c-Met receptor tyrosine kinase. HGF, HGFl and their respective receptors have considerable structural similarities and demonstrate cross-reactivity [27,28,29,30]. Crosstalk of RON and c-Met occurs in various cancer cell lines [31,32], with reciprocal trans-autophosphorylation in response to ligand binding. In addition, HGF/SF-activator proteases have been demonstrated to cleave pro-MSP, an inactive single chain precursor of HGFl [33].

Various cytoplasmic effector molecules have been mapped in downstream signaling and direct context-dependent receptor modification of both the HGF-c-Met and HGFl-RON systems. These include growth factor receptor-bound protein 2 (Grb2) [28], phosphatidylinositol 3-kinase (PI3K) [34] and phospholipase C gamma (PLC-γ) [27]. Whilst biological roles of HGF have been explored extensively in this laboratory [35,36], a role for the HGFl/RON pathway in human wound repair has yet to be clearly elucidated. Understanding this closely related scatter factor pathway will allow us to better characterize the HGF-c-Met axis and its secondary activities in the healing milieu. The current study aimed to examine the expression profile of HGFl in primary human wound tissue and explore the biological functionality of HGFl in an immortalized human keratinocyte cell line. We hypothesize a redundant activity of HGFl in modulation of epidermal wound healing.

## 2. Results

### 2.1. Expression Profile of HGFl/RON in Clinical Wound Samples

**Figure 1:**
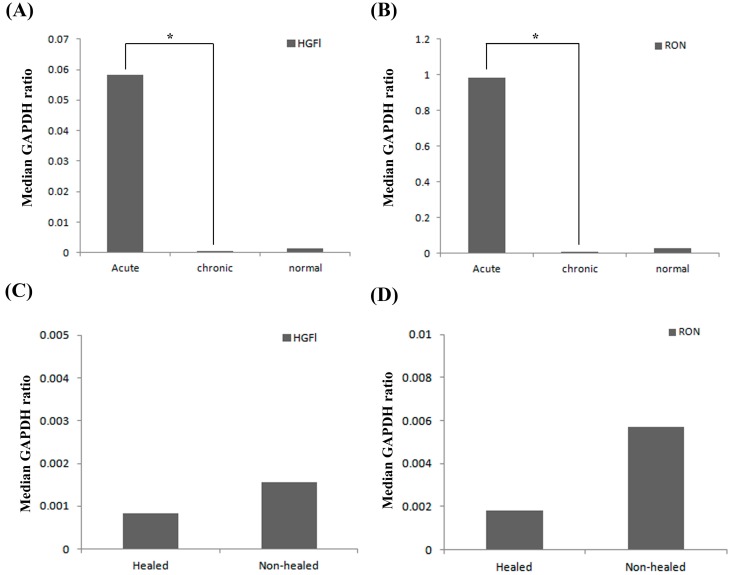
Q-PCR analysis of HGFl and RON transcript expression in clinical wound samples. Expression levels of both HGFl (**A**) and RON (**B**) were significantly elevated in acute wound tissues compared to chronic wound tissues; whereas, similar levels were detected between chronic wound tissues and normal skin. Minimal expression of HGFl (**C**) and RON (**D**) were detected in chronic, healing/non-healing wound tissue, and whilst slightly elevated in non-healing chronic wounds, no significant differences were discovered between the two types of chronic wound tissue for either HGFl or RON. Median GAPDH ratio shown. * represents *p* < 0.05.

The expression pattern of HGFl and RON were explored in the clinical cohorts available in this study. HGFl expression was found to be significantly higher in acute wound tissue (Median 0.058, IQR 0.013–4225) compared to chronic wound tissues (Median 0.0004, IQR 0.0002–0.0068, *p* < 0.05; Figure 1A). Acute wound tissue had higher HGFl expression than normal skin; however, this failed to reach significance. Similarly, no significant difference was observed between normal skin and chronic wound tissues. A similar pattern was observed in RON transcript expression (Figure 1B) where higher levels of transcript were seen in acute wound tissues than in chronic wound tissues and normal skin. Once again, this difference was only significant regarding acute wound tissues (Median 0.986, IQR 0.027–1.54) *vs.* chronic wound tissues (Median 0.0099, IQR 0.003–0.12, *p* < 0.05).

Within the second wound tissue cohort, consistent with the first cohort, relatively low levels of both HGFl (Figure 1C) and RON (Figure 1D) were observed throughout the chronic wound samples (healed or non-healed). No significant differences in either HGFl (*p* = 0.66) or RON (*p* = 0.99) expression were apparent between the chronic healed and chronic non-healed samples.

**Figure 2:**
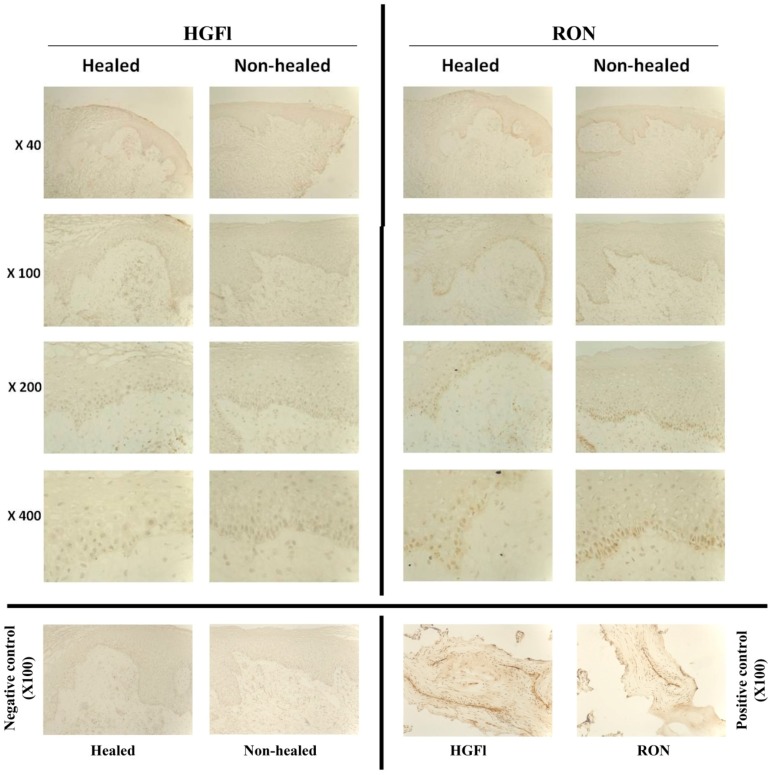
IHC staining for HGFl/RON in chronic healing/non-healing wound tissue. Minimal detection of both HGFl and RON were discovered in both types of chronic wound tissue. No substantial differences were seen in staining intensities of either HGFl or RON between the two types of chronic wound tissues. Negative controls indicate sections stained using secondary antibodies only. Positive controls show placental tissue stained using the respective antibodies. Representative images shown.

In tandem with transcript analysis, immunohistochemical staining of chronic healing and non-healing wound tissues revealed minimal staining for both HGFl and RON (Figure 2). HGFl expression was found to be negative in the epidermis of all tested chronic wound sections. Faint staining for HGFl was observed in the blood vessels of a small proportion of samples, although no difference between healing or non-healing chronic wounds was observed. Minimal staining for RON was observed in the basal nuclear region in many of the chronic wound samples though, again, no differences were observed between the healing and non-healing samples. When the leading wound edge and distal areas of the section towards normal skin were compared, no differences in the localization or intensity of RON staining were observed.

### 2.2. Expression Pattern of HGFl/RON in Human Keratinocytes and Impact on Cell Growth

**Figure 3:**
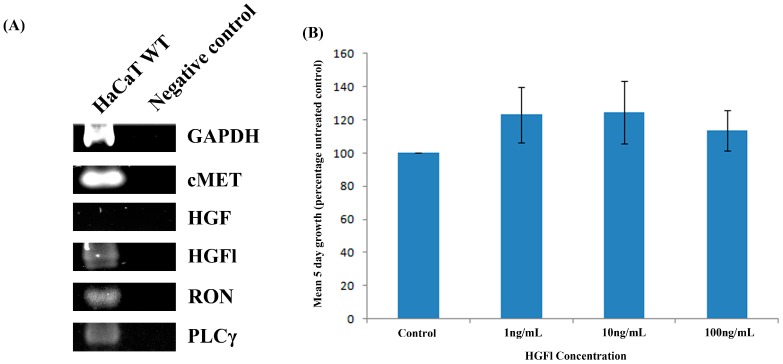
Expression analysis in human HaCaT keratinocytes and impact of HGFl on cell growth rates. HaCaT cells displayed strong expression for the HGF receptor cMET whilst not expressing HGF itself. Moderate expression was observed for HGFl, RON and PLCγ (**A**) Treatment of HaCaT keratinocytes with rhHGFl over a range of concentrations did not significantly impact on cell growth rates (**B**) Representative PCR images or Mean values ± SEM shown.

Transcript analysis of human HaCaT keratinocytes (Figure 3A) demonstrated that this cell line displayed moderate expression of both HGFl and RON. Additionally, it also expressed the HGF receptor cMET at a high level, but not HGF at a detectable intensity. The impact of HGFl on HaCaT cell proliferation was explored through the additional rhHGFl, over a range of concentrations. Treatment with rhHGFl did not significantly impact the five-day growth rates of the HaCaT cell line (*p* > 0.05).

### 2.3. HGFl Impacts on HaCaT Cell Migration

**Figure 4:**
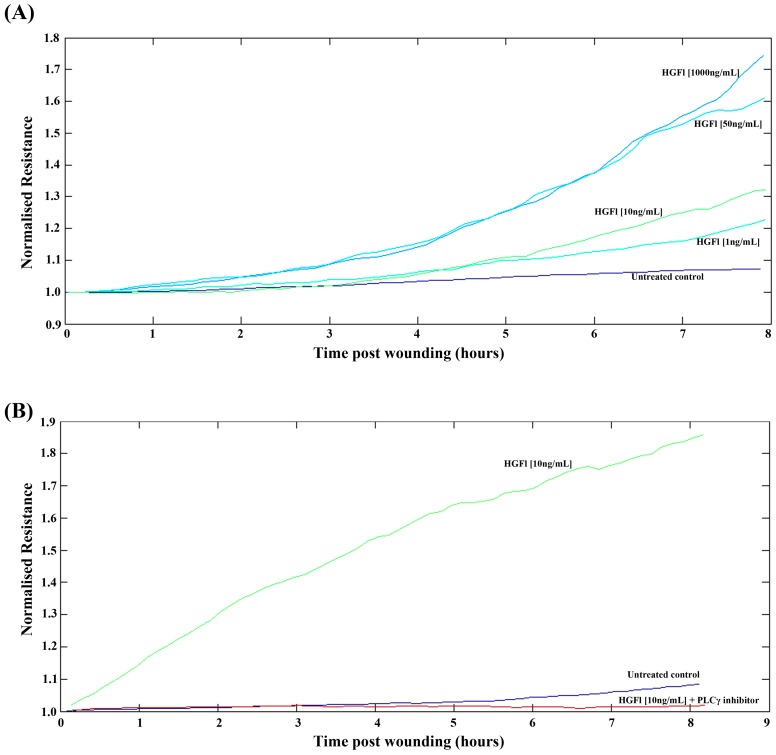
ECIS analysis of the impact of HGFl on HaCaT cell migration. rhHGFl enhanced HaCaT cell migration rates over all concentrations tested, with the strongest response being observed at 50 ng/mL and 1000 ng/mL concentrations (**A**) Inhibition of PLCγ signaling inhibited the pro-migratory response elicited by rhHGFl (**B**). Representative images shown.

The electric cell substrate impedance sensing (ECIS) system was used to detect the impact of rhHGFl on HaCaT cell migration following electrical wounding (Figure 4). Cell migration appeared to be enhanced following the addition of rhHGFl in a dose-dependent manner, with the most significant impact on cell migration being observed at the higher 50 ng/mL and 1000 ng/mL concentrations (Figure 4A). In order to explore potential pathways that may account for these pro-migratory effects of HGFl, a number of small molecule inhibitors were added, in addition to rhHGFl, to explore potential links between HGFl and these other signaling pathways. Of these pathways tested, HGFl appeared to have links with PLCγ signaling pathway. The pro-migratory effect of rhHGFl appeared to be completely stopped through the addition of a small molecule inhibitor to PLCγ, with levels of migration returning to approximately that of untreated control HaCaT cells (Figure 4B).

## 3. Discussion

This paper describes differential HGFl/RON expression in human wound tissue. HGFl/RON transcript expression was significantly greater in the “actively healing” acute wound tissues than in the “chronic” tissues and the relatively quiescent “normal skin” tissues. HGFl/RON expression was generally low in both the “healing” and “non-healing” chronic group. Immunohistochemistry showed low-level HGFl/RON expression in chronic wound samples, with little variation in expression between the chronic healing and non-healing groups. Both HGFl and RON were seen to be expressed in the immortalized human keratinocyte cell line. When HGFl was added to HaCaT cells in an *in vitro* model of wounding, a concentration-dependent pro-migratory effect was observed. The addition of a PLCγ inhibitor reversed the rate of migration to baseline levels at all concentrations of added HGFl. HGFl had no effect on cellular growth.

To the best of our knowledge, this is the first paper to describe differential expression of HGFl in clinical wound samples. Greater expression in acute compared to chronic wound edge tissues suggests a role for HGFl in acute wound healing. The temporal correlation of this upregulation implicates a role for HGFl in re-epithelization. Whilst causality is impossible to prove from these data, it may be that errors in an HGFl-dependent pathway contribute to wound chronicity. The expression of HGFl/RON in both the healing and non-healing chronic wound samples remained considerably below that of the acutely healing cohort. This reinforced a potential role for HGFl in acute wound healing.

The effect of HGFl on an immortalized human keratinocyte cell line was also analyzed. The concentration-dependent pro-migratory effect of HGFl observed suggests a role for HGFl in perpetuating cell migration during epithelization. Our paper supports the findings of Cowin *et al.* 2001 [20] who described increased HGFl expression in a rodent model of acute wound healing. This group co-localized HGFl to a monocyte subtype, with implications to tissue remodeling. Whilst Bezerra *et al.* 1998 [21] reported HGFl to be non-essential in the murine wound healing pathway, we demonstrated a definite augmentation of epithelial migration with the addition of recombinant HGFl and a definite increase in HGFl expression in an acutely healing clinical wound cohort. Wang *et al.* 1996 [23] also found a pro-migratory role of HGFl in a murine *in vitro* model. However, we were unable to replicate reported increases in cell population growth in our human keratinocyte cell line.

The ECIS model used permitted multiple simultaneous experimental repeats with high accuracy estimation of migration distance above that of traditional scratch wound healing models. However, the use of an immortalized cell line for a wound healing study, although well documented by this lab [4,37], may lack translatability. Exploration of the role of HGFl in clinical wound healing may benefit from the use of a primary human keratinocyte cell line.

PLCγ appears to be a key downstream effector via which HGFl retains its pro-migratory effect. Future work is required to substantiate this finding. Wang *et al.* 1996 [23] noted a similar effect with the addition of a phosphatidylinositol-3 kinase inhibitor. Use of other single molecular inhibitors, or combinations of pathway inhibitors, in ECIS wounding models would help better delineate HGFl’s clinical and therapeutic roles. This work is currently ongoing, though the observed relationship between HGFl and PLCγ are of interest, given the role of PLCγ signaling in cell motility, cancer progression and invasion, and its links to HGF-induced cancer invasion and motility [38,39]. Our current data implies that PLCγ may act in a similar fashion as that seen in cancer cells’ responses to HGF, acting to facilitate HGFl pro-migratory effects in keratinocytes. In addition, although alluded to herein, the exploration of the interaction of HGFl with components of the extracellular matrix, including α6β4 integrin and α3β1 integrin, has been overlooked and warrants further investigation.

Epidermal wound healing is a complex and multifactorial process. HGFl is upregulated in acute wound tissue, displaying normal healing in comparison to chronic, non-healing wound tissue. HGFl may have a role in augmenting clinical wound healing through a pro-migratory effect on human epithelial cell lines.

## 4. Materials and Methods

### 4.1. Wound Edge Biopsies

Two cohorts of wound edge tissue were used for analysis, collected from patients in attendance at multidisciplinary wound-healing clinics at the University Hospital of Wales, Cardiff. For the first cohort (South East Wales Research Ethics Committee reference number 04/WSE02/10), chronic wound tissue was obtained from the periphery of chronic leg ulcers of proven venous aetiology that were refractory to six months of standard treatment. Acute wound tissue was obtained from the edge of surgical wounds (excision of pilonidal disease) within 6 weeks of the procedure. Normal skin was taken from the inner aspect of the upper arm of healthy volunteers under local anesthetics. All wounds sampled were deemed non-infected by an overseeing physician. Ten acute samples, 10 normal skin samples and 14 chronic samples were used herein. Details of the patient cohort and biopsy techniques have been described elsewhere [36].

For the second cohort, wound edge tissue from patients with chronic venous leg ulcers were obtained (South East Wales Research Ethics Committee reference number 09/WSE02/59). Wounds were measured at the time of the biopsy, treated as per best medical treatment, and re-measured at 3 months. Static or enlarging wounds were deemed chronic non-healing, whilst those reducing in size were deemed chronic healing. This cohort consisted of 20 healing chronic wounds and 51 non-healing chronic wounds, as described previously [37].

Core biopsies were stored initially at −80 °C, before submersion in liquid nitrogen for storage prior to analysis. They were then sectioned on a Leica cyrostat (Leica Microsystems (UK) Ltd., Milton Keynes, UK) to a thickness of 7 µm for immunohistochemical staining and 20 µm for RNA extraction (multiple sections), reverse transcription and quantitative polymerase chain reaction (Q-PCR) transcript analysis.

### 4.2. Materials for Cell Culture

All materials and reagents were purchased from Sigma–Aldrich (Poole, UK), unless otherwise specified. The immortalized human keratinocyte cell line “HaCaT” (German Cancer Institute, Heidelberg, Germany) was used herein. Cells were cultured in DMEM/Ham’s F12 with l-Glutamine medium, supplemented with added amphotericin B, streptomycin, penicillin and 10% fetal calf serum. Recombinant human HGFl (rhHGFl) was obtained from R&D systems (Abingdon, UK). The biological functionality of HGFl was interrogated with the addition of rhHGFl over a range of concentrations and compared to an untreated control. PLCγ inhibitor (U73122) was purchased from Calbiochem (Merck Chemicals Ltd., Nottingham, UK).

### 4.3. RNA Extraction, Reverse Transcription Polymerase Chain Reaction (RT-PCR) and Quantitative RT-PCR

Cells were lysed in TRI reagent, and multiple sections of wound tissue were homogenized using a handheld homogenizer (Cole Palmer, London, UK) in ice-cold TRI reagent. RNA extraction was performed on both cells and wound tissue as per manufacturer’s guidelines. Extracted RNA was re-suspended in DEPC water, quantified using a spectrophotometer (WPA UV 1101, Biotech, Cambridge, UK) and standardized. cDNA was generated via reverse transcription of the RNA template using a High-Capacity cDNA Reverse Transcription Kit (Life Technologies, Paisley, UK). Polymerase chain reaction (PCR) was performed in a T-Cy Thermocycler (Creacon Technologies Ltd., Emmen, The Netherlands) using GoTaq master mix (Promega, Hampshire, UK) under the following conditions: initial denaturing (94 °C, 5 min), followed by 32–34 cycles of denaturing, annealing and elongation (94 °C for 30 s, 55 °C for 40 s, 72 °C for 50 s), and final extension at 72 °C for 10 min before holding at 4 °C. Details of primers used are in Table 1. PCR products were loaded onto an agarose gel, electrophoretically separated, stained in SYBR Safe and visualized under blue light. Images were obtained using a camera mounted to a U-Genius 3 Gel documentation system (Syngene, Cambridge, UK). For the cohort tissue samples Q-PCR was performed. The Q-PCR assay used the Amplifluor (TM) detection system (Intergen Inc., New York, NY, USA) as previously described [40,41]. cDNA was added to Hotstart Q-master mix (AbGene), 10 pmol forward primer, 1 pmol reverse primer (containing the z sequence) and 10 pmol of the FAM-tagged universal Z probe (Intergen Inc., New York, NY, USA). Primer sequences used are detailed in Table 1. Cycle thresholds and conditions used replicated those previously described [6]. An internal standard was simultaneously amplified with the samples to allow a transcript copy number to be calculated using the StepOne™ software version 2.2.2 (Life Technologies, Paisley, UK).

**Table 1:** Primers used for polymerase chain reaction (PCR) and quantitative polymerase chain reaction (Q-PCR) (*ACTGAACCTGACCGTACA* is the z sequence).

Primer	Forward	Reverse
HGFl	AGGTGCAGTTTGAGAAGTGT	CTGTGTCATTACCCGTACCT
RON	CATCCACCCAGTGCCAAC	*ACTGAACCTGACCGTACA*CCACACAGTCAGCCACAG
cMET	*ACTGAACCTGACCGTACA*GAGCCAAAGTCCTTTCAT	ATCGAATGCAATGGATGAT
HGF	TACTGCAGACCAATGTGCTA	*ACTGAACCTGACCGTACA*GCATTGTTTTCTCGCTTTAT
PLCγ	AGAACGACATCAGCAACTCT	GCATATGAGTTGGGTTCATT
GAPDH (PCR)	AGCTTGTCATCAATGGAAAT	CTTCACCACCTTCTTGATGT
HGFl	GACCAGGCGCCATCAATC	*ACTGAACCTGACCGTACA*CTTGGAACGCCGCTGATC
GAPDH (Q-PCR)	CTGAGTACGTCGTGGAGTC	*ACTGAACCTGACCGTACA*CAGAGATGATGACCCTTTTG

### 4.4. Immunohistochemical Staining of Wound Tissue

Serial frozen sections of wound tissue were fixed in dried acetone (Fisher, UK) before being hydrated in tris buffered saline (TBS) wash buffer and incubated in a 10% horse serum wash buffer for one hour. Anti-HGFL (R&D 352-MS) or anti-RON (R&D AF691) primary antibodies were added to the sections at a dilution of 1:100 and incubated for 1 h. The sections were then washed in TBS wash buffer 4 times and antibody localization was identified with a standard streptavidin-biotin peroxidase technique (Vector Laboratories, Peterborough, UK). The final reaction product was developed with 3,3'-diaminobenzidine (DAB), and then the sections were counterstained with Mayer’s haematoxylin (Merck, Germany), washed in running tap water, dehydrated through a graded series of alcohol, cleared in xylene, mounted in DPX mounting medium (Merck, Germany) and observed under a microscope. Any positive staining was seen as a brown-black deposit, whilst negative areas were identified as blue counterstained nucleated cells.

### 4.5. In Vitro Growth Assay

Three thousands cells were seeded into each well of two 96-well plates with or without the addition of HGFl at a defined concentration. The plates were incubated at 37 degrees for 1 or 5 days as appropriate. At their respective end-points the cells were fixed with 4% formaldehyde (*v*/*v*), stained with 0.5% (*w*/*v*) crystal violet solution and treated with 10% acetic acid (*v*/*v*). Subsequently, the solution was analyzed using a 96 well plate spectrophotometric Bio-Tek ELx800177 multi-plate reader (Bio-Tek, Winooski, VT, USA) at 540 nm wavelength and cell density calculated indirectly based on absorbance.

### 4.6. Electric Cell–Substrate Impedance Sensing Analysis of Cellular Migration

The electric cell–substrate impedance sensing (ECIS) system (Applied Biophysics, Troy, NJ, USA) was also used to analyze HaCaT migration, using methods previously described [42]. Eighty thousands (80,000) cells were seeded in HEPES (4-(2-hydroxyethyl)-1-piperazineethanesulfonic acid) buffered medium, with or without the respective HGFl treatment, and incubated until a confluent monolayer had been formed. The monolayer was wounded electrically using the manufacturer’s “wound” function. As cells migrated back towards the electrode, a change in cell-substrate resistance was detected. This rate of change was measured using the ECIS software provided, providing a surrogate for rate of migration.

### 4.7. Statistical Analysis

Statistical analysis was undertaken using the Sigmaplot 11 statistical package (Systat Software Inc., London, UK). Data was compared using Mann Whitney, ANOVA on RANKS or ANOVA statistical tests depending on data normality. A minimum number of three repeats for experimental data were preformed. A value of *p* < 0.05 was taken as statistically significant.
